# Supplementary material for: Chemical tuning of spin clock transitions in molecular monomers based on nuclear spin-free Ni(ii)
Source: Chem Sci. 2021 Feb 25;12(14):5123–33. doi: 10.1039/d0sc05856d (PMC8179637; doi:10.1039/d0sc05856d)
Supplement: SC-012-D0SC05856D-s001 [file SC-012-D0SC05856D-s001.pdf]

Electronic Supplementary Material (ESI) for Chemical Science.
This journal is © The Royal Society of Chemistry 2021

Electronic Supporting Information for

Chemical tuning of spin clock transitions in molecular monomers based on nuclear spin-free Ni(II)

Marcos Rubín-Osanz,[a] François Lambert,[b] Feng Shao,[b] Eric Rivière,[b] Régis Guillot,[b] Nicolas Suaud,[c] Nathalie Guihéry,[c] David Zueco,[a] Anne-Laure Barra,[d] Talal Mallah,*[b] and Fernando Luis *[a]

1. Supplementary information

1.1. Synthesis

N-méthyl N,N-bis{3-[(2-imidazolyl-méthyl)-amino]-propyl}- amine (2-imdipa): 1.09 g (0.0075 mol) of methy-di(3-aminopropyl)-amine are dissolvel in20 ml of methanol. The solution is added dropwise under stirring to 1.5 g (0.0156 mol) of 2-carboxaldehyde imidazole dissolved in 45 ml of hot methanol. The obtained solution is refluxed (70 °C) for 15 minutes and left to cooldown to room temperature. 300 mg of activated Pd/C (10% Pd) are added very slowly to the solution. The mixture is loaded in a steel vessel and left to stirr at room temperature overnight under a H_2 pressure of 40 bars. The Pd/C is removed by filtration on cellite and thoroughly washed with methanol. The solution is evaporated under vacuum to give a yellow oil, with a yield of 98%. NMR 1H ($CDCl_3$): 1.55 ppm (m, 4H), 2,1 ppm (s, 3H), 2,3 ppm (t, 4H); 2,6 ppm (t, 4H) ; 3,85 ppm (s, 4H) ; 6-6,5 ppm (s large, 4H) ; 6,9 ppm (s, 4H).

[Ni(2-imdipa)(NCS)][NCS]•(0.5MeOH) (4) : 6.1 g of 2-imdipa (0.02 mol) are dissolved in 100 ml of methanol and added to a 100 ml methanolic solution containing 6.4 g (0.02 mol) of $[Ni(H_2O)_6]Cl_2$. The solution is stirred for 15 minutes at room temperature to which is added, under stirring, 200 ml of methanol containing 6.8 g (0.08 mol) of NH_4NCS. A change of color from blue-green to violet is observed. The solution is reduced to a volume of 150 ml under vacuum and 100 ml of water are added. The solution is then left to evaporate for three days. Blue-violet crystals form, they are collected by filtration washed within a minimum of distilled water and left to dry. The crystals are dissolved in hot methanol (150 ml) and filtered to eliminate a white precipitate. Yield 3 g (30%). Small crystals suitable for single crystal X-ray diffraction are obtained by ether diffusion into a methanolic solution of the complex. Large crystals (5x3x3 mm^3) are obtained by using the small crystals as seeds in a saturated methanolic solution of the complex under ether diffusion. Elemental analysis performed on microcystalline powder give for $NiC_{17.5}H_{29}N_{14}O_{0.5}S_2$ (Mw = 495.83) exp (calc.) %Ni: 11.41 (11.83), %C: 41.04 (42.35), %H:5.83 (5.85), %N: 25.78 (25.41), %S: 12.93 (13.01).

1.2. Crystallographic characterization of complex 4

X-ray diffraction data for compound **4** were collected on a Kappa X8 APPEX II Bruker diffractometer equipped with a graphite-monochromated Mo_{K}[illegible] radiation (λ = 0.71073 Å). Crystal was mounted on a CryoLoop (Hampton Research) with Paratone-N (Hampton Research) as cryoprotectant and then flashfrozen in a nitrogen-gas stream at 100 K. The temperature of the crystal was maintained at the selected value by means of a 700 series Cryostream cooling device to within an accuracy of ±1 K. The data were corrected for Lorentz polarization and absorption effects. The structures were solved by direct methods using SHELXS-97 [1] and refined against F^2 by full-matrix least-squares techniques using SHELXL-2018 [2] with anisotropic displacement parameters for all non-hydrogen atoms. Hydrogen atoms were located on a difference Fourier map and introduced into the calculations as a riding model with isotropic thermal parameters. All calculations were performed by using the Crystal Structure crystallographic software package WINGX [3].
The crystal data collection and refinement parameters are given in Table S1.

The asymmetric unit of **4** consists of two independent molecules of [Ni(2-Imdipa)](NCS), two (NCS) and only one methanol.
CCDC 2000847 contains the supplementary crystallographic data for this paper. These data can be obtained free of charge from the Cambridge Crystallographic Data Centre via http://www.ccdc.cam.ac.uk/Community/Requestastructure.

1.3. Symmetry operations and magnetic field orientation for crystals of complex 4

The four molecule orientations in the unit cell of **4** are related by an inversion, a 2-fold screw axis along $\hat{\boldsymbol{b}}$ and a glide plane generated by $\hat{\boldsymbol{a}}$ and $\hat{\boldsymbol{c}}$. Translations are irrelevant, so we can consider the 2-fold screw axis as a 2-fold rotation axis and the glide plane as a mirror plane. We ask now how a vector $\vec{\boldsymbol{x}} = (\boldsymbol{x_1}, \boldsymbol{x_2}, \boldsymbol{x_3})$ transforms under these symmetry elements. If $\boldsymbol{x_1}, \boldsymbol{x_2}, \boldsymbol{x_3}$ are the coordinates of $\vec{\boldsymbol{x}}$ along $\hat{\boldsymbol{a}}$, $\hat{\boldsymbol{b}}$ and $\hat{\boldsymbol{a}} \times \hat{\boldsymbol{b}}$:

- Inversion:

$$\boldsymbol{i}\vec{\boldsymbol{x}} = \begin{pmatrix} -1 & 0 & 0 \\ 0 & -1 & 0 \\ 0 & 0 & -1 \end{pmatrix} \begin{pmatrix} x_1 \\ x_2 \\ x_3 \end{pmatrix} = -\vec{\boldsymbol{x}}$$

- 2-fold rotation axis along $\hat{\boldsymbol{b}}$:

$$\boldsymbol{C_2}\vec{\boldsymbol{x}} = \begin{pmatrix} -1 & 0 & 0 \\ 0 & 1 & 0 \\ 0 & 0 & -1 \end{pmatrix} \begin{pmatrix} x_1 \\ x_2 \\ x_3 \end{pmatrix} = \begin{pmatrix} -x_1 \\ x_2 \\ -x_3 \end{pmatrix}$$

- Mirror plane generated by $\hat{\boldsymbol{a}}$ and $\hat{\boldsymbol{c}}$ = mirror plane generated by $\hat{\boldsymbol{a}}$ and $\hat{\boldsymbol{a}} \times \hat{\boldsymbol{b}}$:

$$\boldsymbol{\sigma}\vec{\boldsymbol{x}} = \begin{pmatrix} 1 & 0 & 0 \\ 0 & -1 & 0 \\ 0 & 0 & 1 \end{pmatrix} \begin{pmatrix} x_1 \\ x_2 \\ x_3 \end{pmatrix} = \begin{pmatrix} x_1 \\ -x_2 \\ x_3 \end{pmatrix}$$

The magnetic field $\vec{\boldsymbol{H}}$ contributes to the Hamiltonian through the Zeeman term:

$$\mathcal{H}_{Zeeman} = -\vec{\boldsymbol{\mu}} \cdot \vec{\boldsymbol{H}}$$

Where $\vec{\boldsymbol{\mu}}$ is the magnetic moment of the ion. The magnetic moments of the four molecule orientations are related through the symmetry operations described above. Then we have four different Zeeman terms:

- Inversion:

$$\mathcal{H}_{Zeeman} = -(\boldsymbol{i}\vec{\boldsymbol{\mu}}) \cdot \vec{\boldsymbol{H}} = +\vec{\boldsymbol{\mu}} \cdot \vec{\boldsymbol{H}}$$

- 2-fold rotation axis along $\hat{\boldsymbol{b}}$:

$$\mathcal{H}_{Zeeman} = -(\boldsymbol{C_2}\vec{\boldsymbol{\mu}}) \cdot \vec{\boldsymbol{H}} = \mu_1 H_1 - \mu_2 H_2 + \mu_3 H_3$$

- Mirror plane generated by $\hat{\boldsymbol{a}}$ and $\hat{\boldsymbol{c}}$ = mirror plane generated by $\hat{\boldsymbol{a}}$ and $\hat{\boldsymbol{a}} \times \hat{\boldsymbol{b}}$:

$$\mathcal{H}_{Zeeman} = -(\boldsymbol{\sigma}\vec{\boldsymbol{\mu}}) \cdot \vec{\boldsymbol{H}} = -\mu_1 H_1 + \mu_2 H_2 - \mu_3 H_3$$

If only a global sign changes, the energy levels and states are the same. This happens if $\boldsymbol{\mu_2} = \mathbf{0}$ (the plane generated by *a* and *c*) or if $\boldsymbol{\mu_1} = \boldsymbol{\mu_3} = \mathbf{0}$ (the cell *b* axis). In these situations, the cosine directors of $\vec{\boldsymbol{H}}$ in the molecular axes ($\vec{\boldsymbol{x}} \cdot \vec{\boldsymbol{H}}$, where $\vec{\boldsymbol{x}}$ is any molecular axis) are also the same (but a global sign). From all these considerations we conclude that we only need to consider one molecule orientation if we measure along the cell b axis or the plane perpendicular to it.

2. Supplementary Tables

Table S1. Crystallographic data and structure refinement details of complex **4**.

**Compound**	**4**
CCDC	2000847
Empirical Formula	2(C_{16} H_{27} N_8 Ni S), 2(C N S), C H_4 O
M_r	992.61
Crystal size, mm^3	0.34 0.29 0.18
Crystal system	monoclinic
Space group	P $2_1/n$
a, Å	14.8018(4)
b, Å	16.4349(4)
c, Å	19.1348(5)
α, °	90
β, °	102.4940(10)
γ, °	90
Cell volume, $Å^3$	4544.6(2)
Z ; Z’	4 ; 1
T, K	100(1)
Radiation type; wavelength Å	MoKα; 0.71073
F_{000}	2088
μ, mm^{-1}	1.064
range, °	1.584 - 32.858
Reflections collected	145 386

Reflections unique	16 791
R_{int}	0.0300
GOF	1.060
Refl. obs. $I > 2(I)$	14 103
Parameters	545
wR_2 (all data)	0.1084
R value $I > 2(I)$	0.0368
Largest diff. peak and hole ($e^{-}.Å^{-3}$)	1.943 ; -0.925

Table S2: Comparison of the bond lengths (in Å) and the angles (in °) of the two independent molecules:

Complex	**Ni(1)**	**Ni(2)**
Ni - N1	2.0980(12)	2.1136(12)
Ni - N2	2.1382(13)	2.1457(13)
Ni - N3	2.1846(13)	2.1760(13)
Ni - N4	2.1279(12)	2.1311(12)
Ni - N5	2.0606(12)	2.0803(12)
Ni - N6	2.0594(14)	2.0605(14)
N1 -Ni - N2	79.66(5)	78.38(5)
N1 -Ni - N3	174.40(5)	174.71(5)
N1 -Ni - N4	91.69(5)	93.11(5)
N1 -Ni - N5	90.59(5)	89.07(5)
N1 -Ni - N6	87.36(5)	88.14(5)
N2 - Ni - N3	96.92(5)	97.20(5)
N2 - Ni - N4	169.20(5)	170.55(5)
N2 - Ni - N5	92.88(5)	95.23(5)

N2 - Ni - N6	89.64(6)	88.94(5)
N3 - Ni - N4	92.19(5)	91.50(5)
N3 - Ni - N5	94.03(5)	94.26(5)
N3 - Ni - N6	88.18(5)	88.88(5)
N4 - Ni - N5	80.67(5)	80.38(5)
N4 - Ni - N6	96.45(5)	94.94(5)
N5 - Ni -N6	176.42(5)	174.42(5)

Table S3. Contribution to *D* and to *E* of the 9 triplet and the 15 singlet excited states for complex **4** determined from *ab initio* calculations

State	Contribution to *D*	Contribution to *E*
T0	-	-
T1	-34.809	-0.868
T2	16.805	5.163
T3	14.84	-4.708
T4	-0.014	-0.018
T5	0.008	0.005
T6	0.002	0.005
T7	0.002	0.001
T8	-0.001	-0.001
T9	0	0
S0	0.001	-0.003
S1	-0.002	0.002
S2	13.188	0.063
S3	-5.882	3.68
S4	-6.769	-3.817
S5	-0.002	-0.005
S6	-0.021	0.001
S7	-0.001	0.025
S8	0.009	-0.002
S9	-0.021	-0.022
S10	-0.37	0.309
S11	-0.328	-0.41
S12	-0.286	0.196
S13	0.962	-0.023
S14	0	0

3. Supplementary Figures

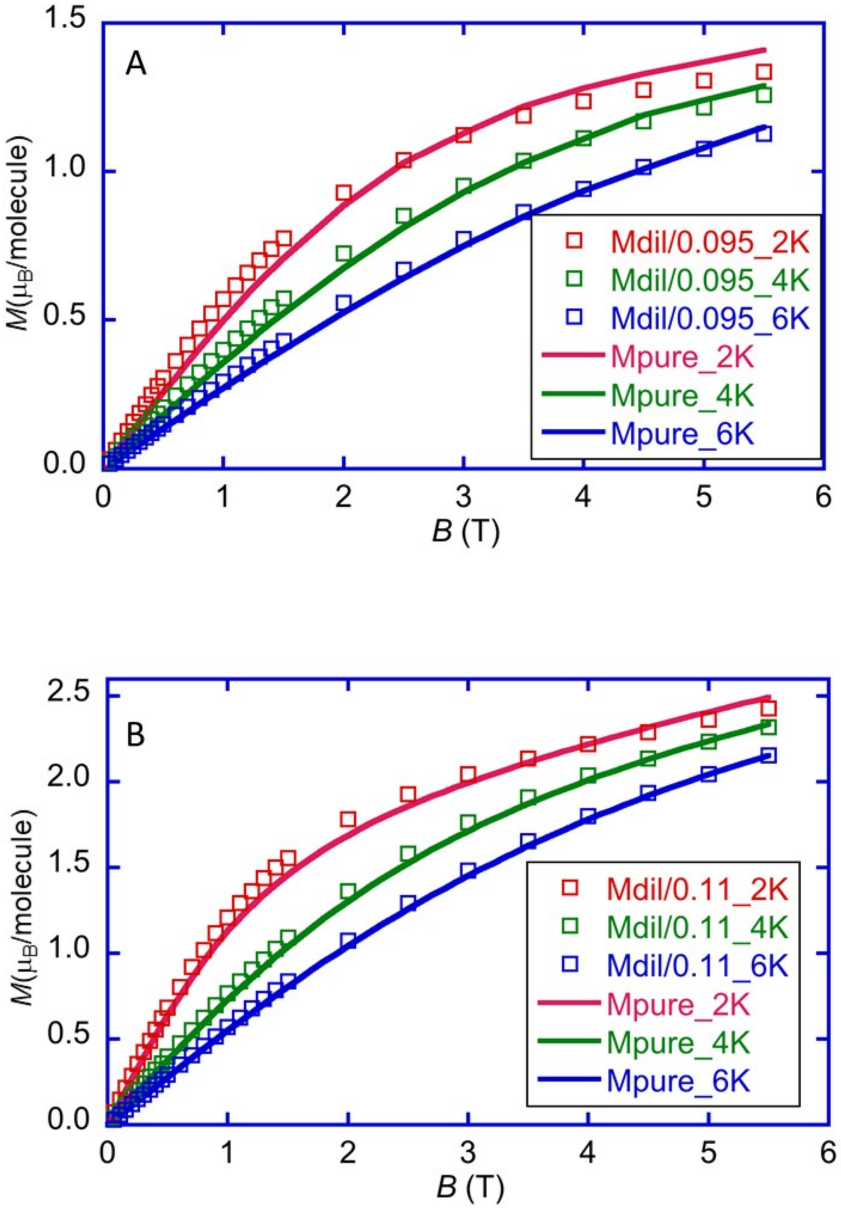

Figure S1. A: Magnetization isotherms measured on powder samples of **1** (lines) and of the magnetically diluted $\mathbf{1}_{d9.5\%}$ sample (symbols) at the indicated temperatures. The scaling of the former data shows that the actual concentration is close to 9.5%. B: Magnetization isotherms measured on powder samples of **2** (lines) and of the magnetically diluted $\mathbf{2}_{d11\%}$ sample (symbols) at the indicated temperatures. The scaling of the former data shows that the actual concentration is close to 11%.

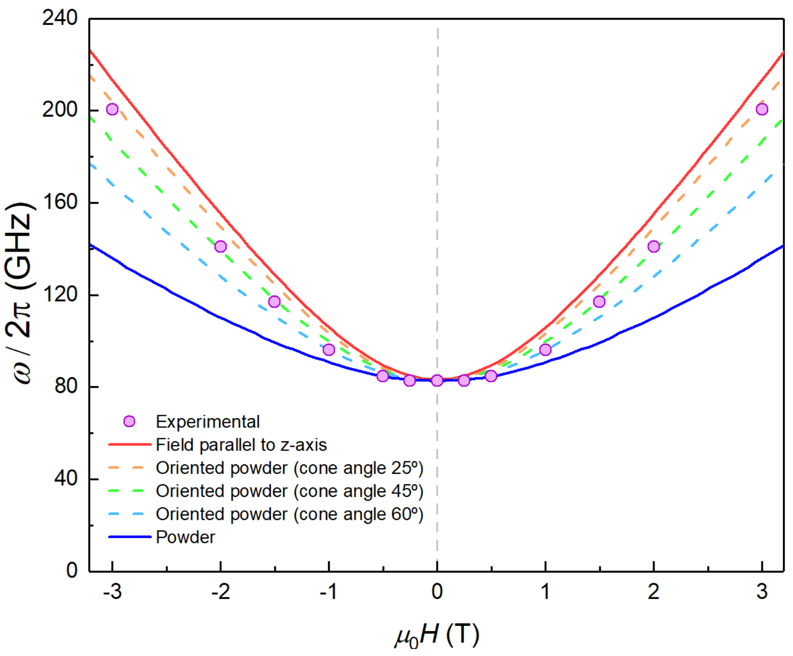

Figure S2. Experimental effective energy gap $\hbar\omega = k_B T_0/0.42$, with T_0 the field-dependent temperature of the heat capacity maximum measured on a powdered sample of **1** (Fig. 3 of the main text). Solid lines show the behaviour simulated for easy axes spread within a cone with varying aperture θ. The limits $\theta = 0$ and $\theta = \pi/2$, shown in Fig. 3 of the main text, correspond to the anisotropy axes being perfectly aligned along the magnetic field and being randomly oriented, respectively.

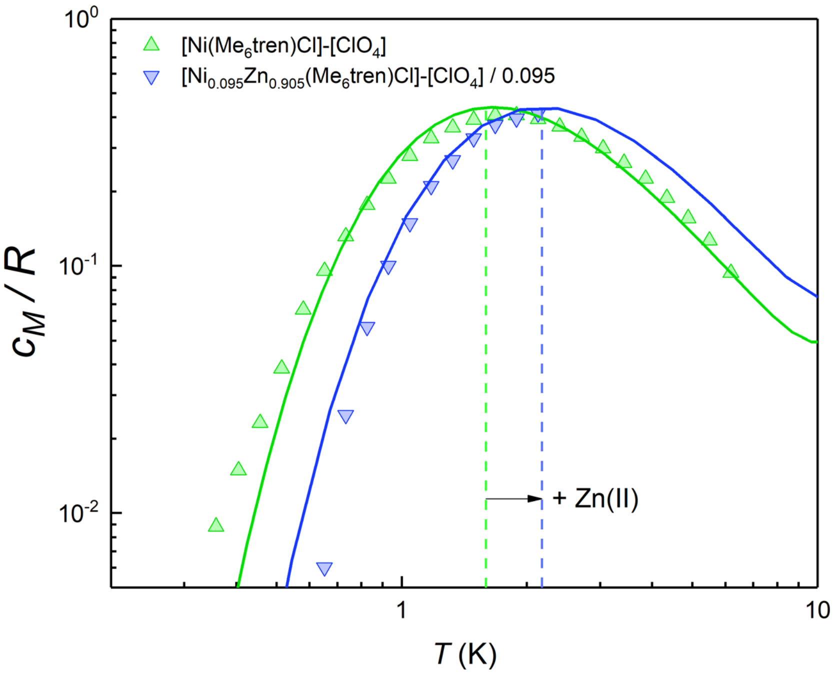

Figure S3. Specific heat of complexes **1** and **1**$_{\mathbf{d9.5\%}}$ at $\boldsymbol{\mu_0 H = 0}$. The lattice contribution has been subtracted, and the remaining magnetic contribution is normalised per mol of magnetic molecules. Despite the expected decrease in the strength of intermolecular magnetic interactions, these data show the same Schottky anomaly, even shifted to slightly higher temperatures. Therefore, the anomaly is due to the zero field splitting generated by the ligand field of each individual molecule.

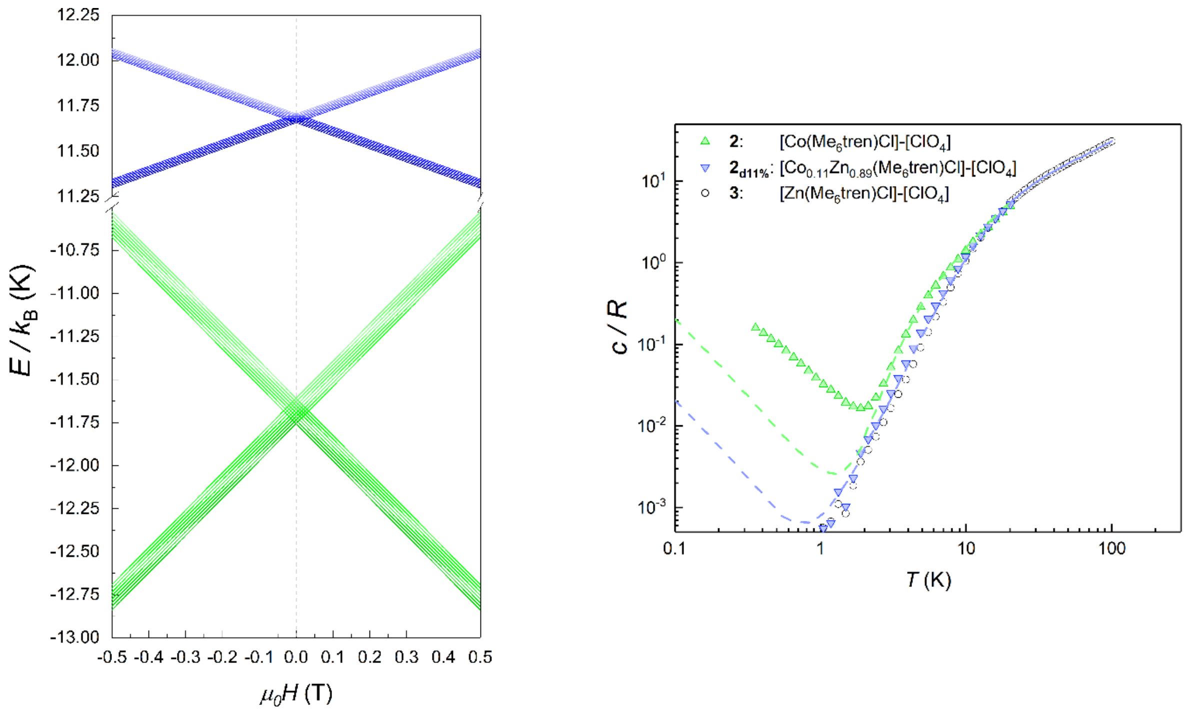

Figure S4. Left: Level structure of an isolated molecule of complex **2** including hyperfine interactions with a coupling constant A_{hf}/k_B = 14 mK (see [4] and references therein) between the S = 3/2 electronic spin and the I = 7/2 nuclear spin. Right: The hyperfine level splitting gives rise to an additional contribution to the specific heat, which is shown by the dashed lines and compared to the experimental data obtained for the pure sample **2** and the magnetically diluted sample $\mathbf{2}_{11\%}$. This comparison confirms that the hyperfine contribution cannot account for the specific heat of **2** below 1 K.

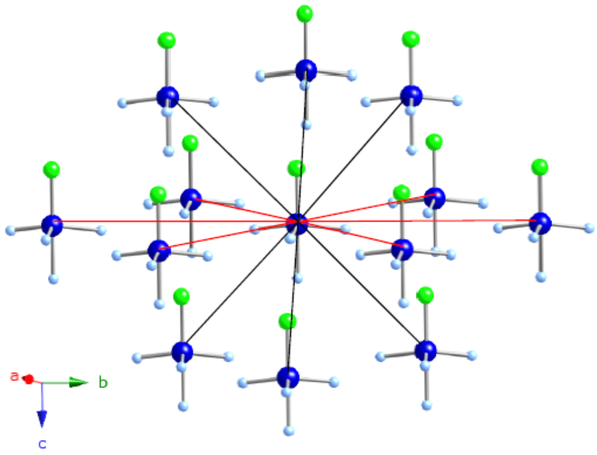

Figure S5. Location of the 12 nearest neighbour molecules of each [Co(Me$_6$tren)Cl]-(ClO_4) molecule in its crystal lattice, taken from reference [4]. This lattice has been used for the Monte Carlo calculations of spin-spin interactions, whose results are shown in Fig. 4 of the main text and in Fig. S4, and for the quantum "toy model" calculations shown in Fig. S6 below.

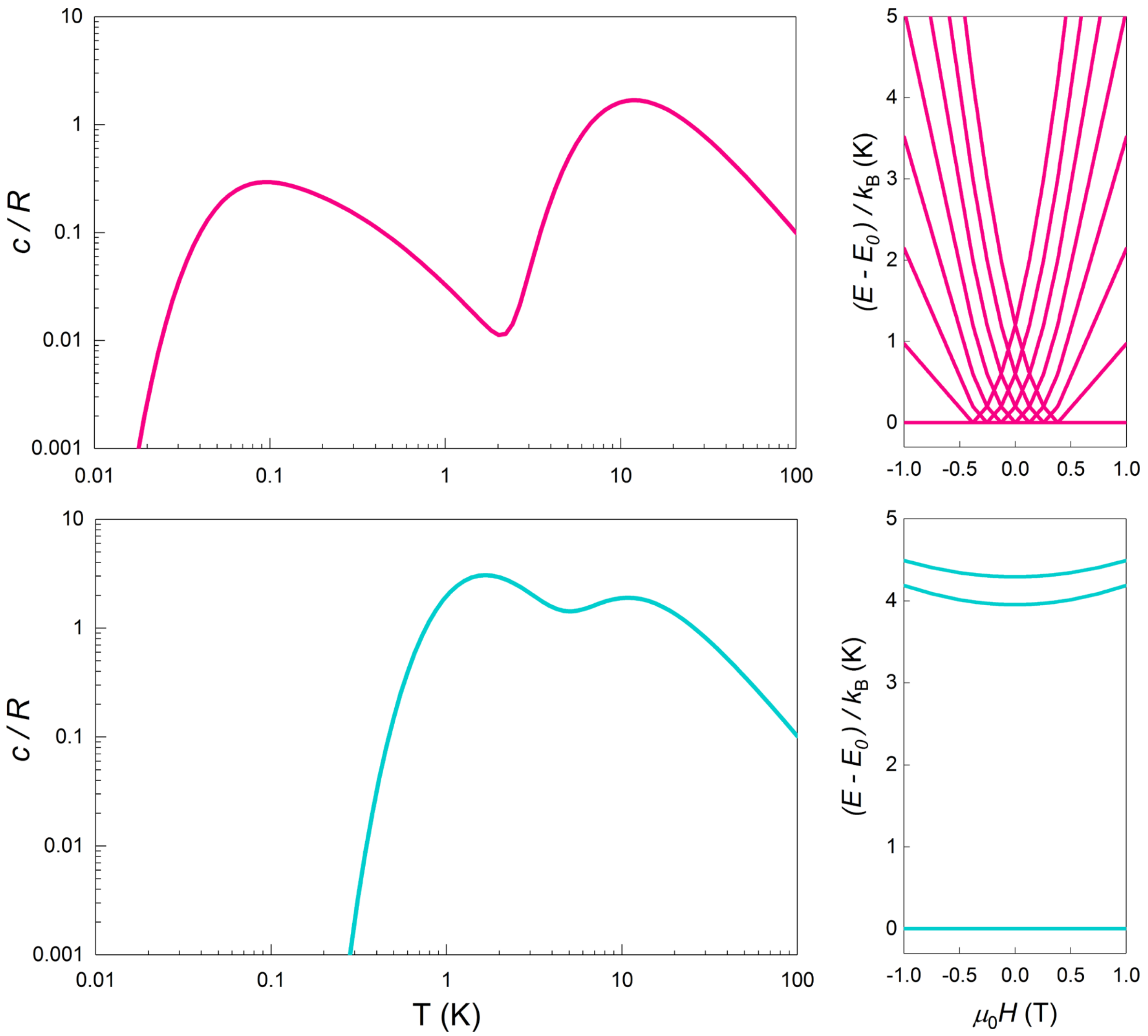

Figure S6. Results of quantum calculations performed on a set of 6+1 S = 1 spins located in the lattice shown in Fig. S5 and that include the central molecule plus its six nearest neighbours in the same crystal plane. The results have been obtained by numerical diagonalization of the multiple spin Hamiltonian, which includes the single molecule anisotropy (as given by Eq. (1) in the main text) and the couplings between different molecular spins (as given by Eq. (4) in the main text with J = -0.035 cm^{-1} as determined for complex **2**). Panels at the top show the specific heat (left) and the field-dependent energy level structure (right, with energies referenced to the ground level) for the case of a vanishingly small quantum gap Δ. The bottom panels show the calculations for Δ = 2.9 cm^{-1}, as found for complex **1**. The presence of this gap lifts up in energy excitations due to spin-spin interactions and "clears up" their contribution to c/R at very low temperature, just as it is observed in the experimental data measured on **1** (Fig. 2 of the main text).

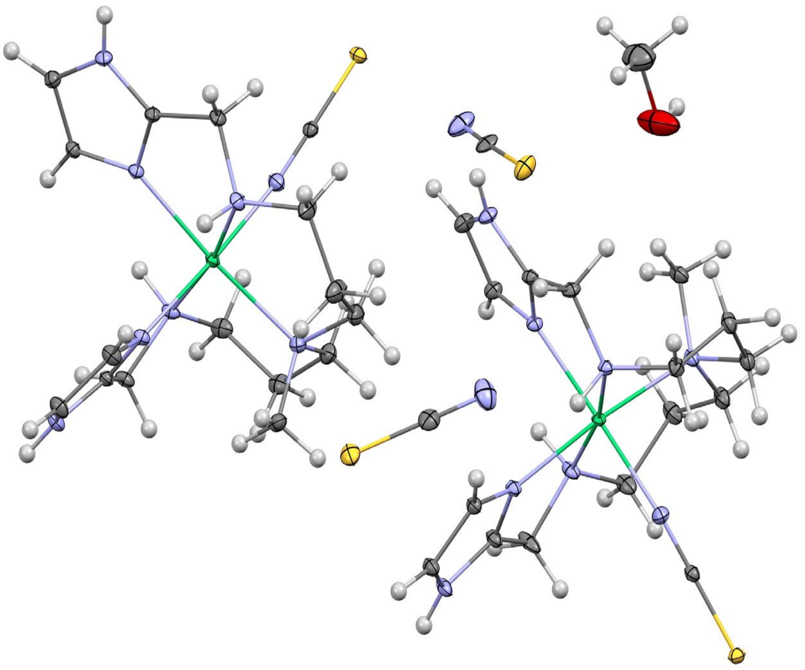

Figure S7. An ORTEP drawing of compound **4**. Thermal ellipsoids are shown at the 30% level.

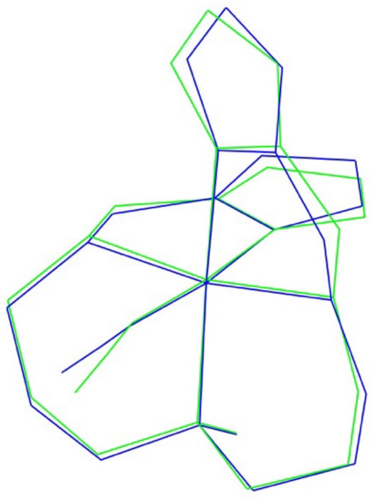

Figure S8. Schematic view of a superposition of the two crystallographically independent molecules

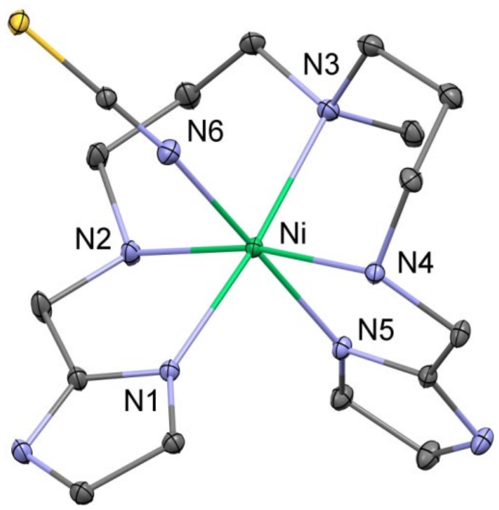

Figure S9. ORTEP drawing of complex **4** with atom numbering, H atoms are omitted for clarity

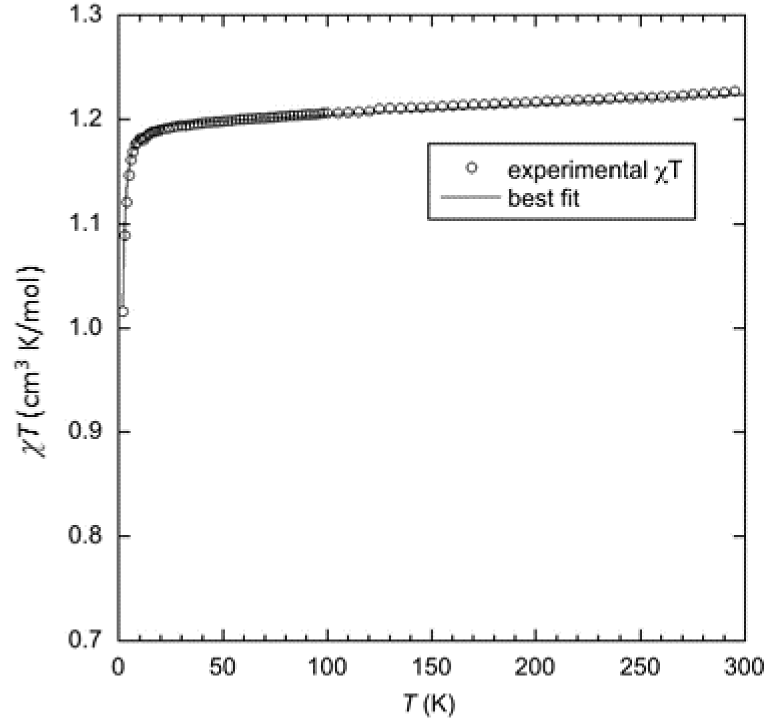

Figure S10. Thermal variation of χT (○) experimental data and (—) best fit (see main text).

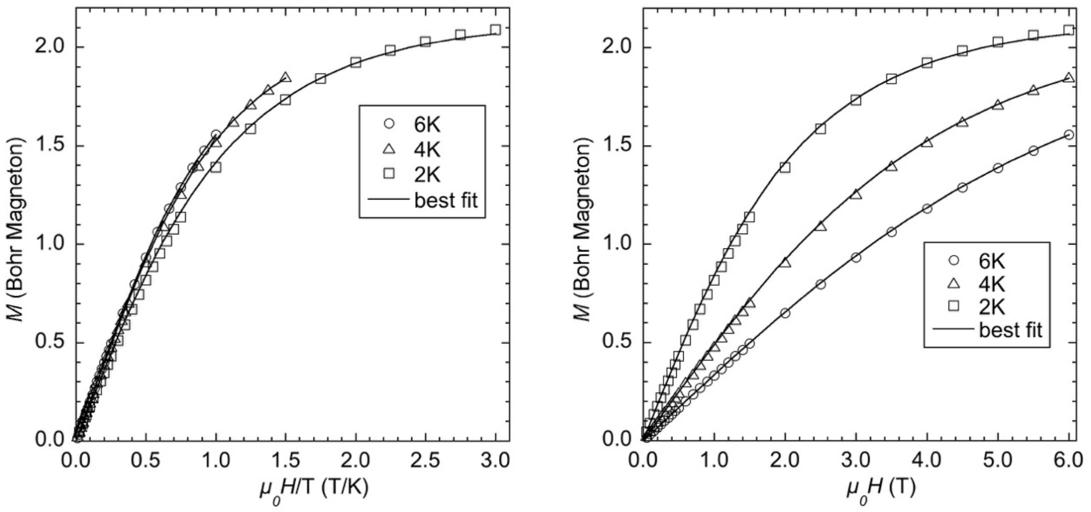

Figure S11. Magnetization (M) isotherms of a powder sample of **4** measured at T = 6 (□), 4 (△) and 2(○) K *versus* μ_0H/T (left) and *versus* μ_0H (right), (—) best fit (see main text).

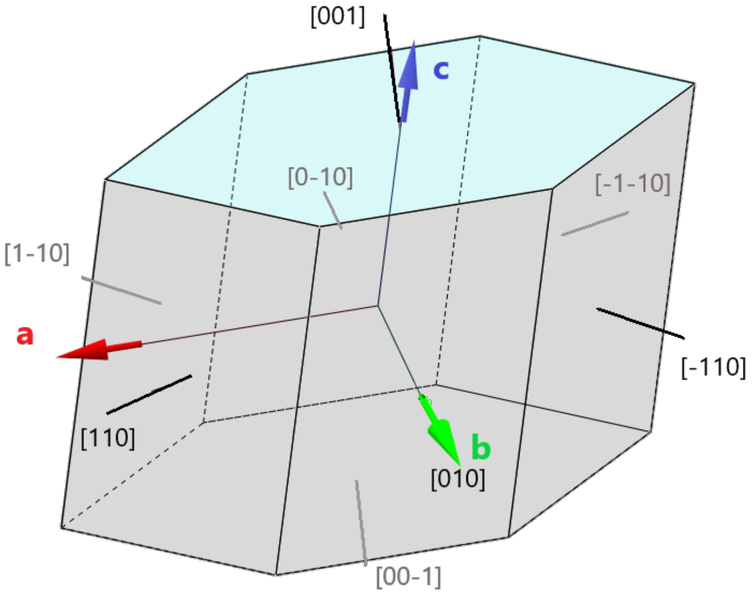

Figure S12. Schematic view of a single crystal of **4** with the crystallographic planes (001), (00-1), (010), (0-10), (110), (-1-10), (-110) and (1-10), which were modelled computationally using the crystal morphology editor/viewer (KrystalShaper) software (version 1.5.0 for Windows [5]) and the lattice parameters of **4**.

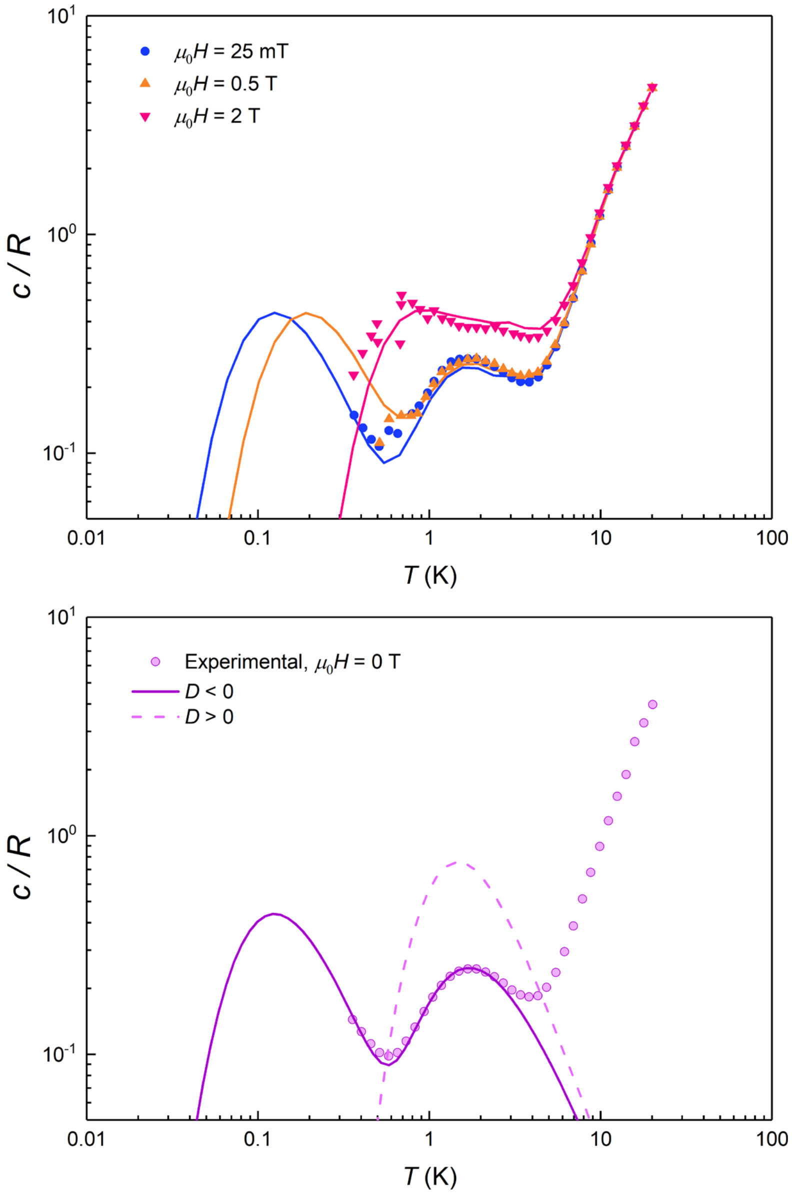

Figure S13. Top: Specific heat of a single crystal of **4** measured for magnetic fields 0.025 T (blue), 0.5 T (orange) and 2 T (pink) applied along the cell *b* axis. Solid lines show simulations performed for a magnetic field parallel to the magnetic *y* axis with the magnetic anisotropy parameters given in the main text. The agreement between experiment and theory shows that the molecular *y* axis points along the cell *b* axis. Bottom: Comparison between the zero-field specific heat data with simulations performed for positive and negative *D*, as indicated. The results unequivocally show that the magnetic anisotropy in **4** has a uniaxial character ($D < 0$).

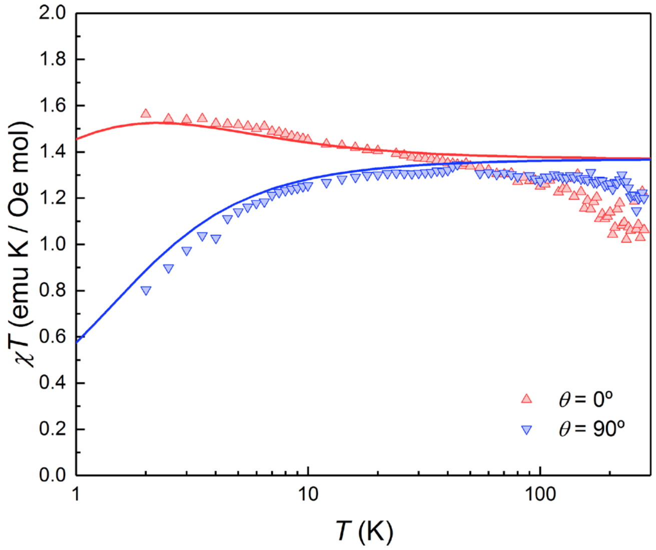

Figure S14. Magnetic susceptibility of a single crystal of **4** measured with the magnetic field applied along the orientations that give rise to a maximum (red) and a minimum (blue) magnetization in Figure 9 of the main text. Solid lines show simulations for the magnetic field applied along the *a* (red) and the *b* (blue) crystal axes, with the magnetic anisotropy parameters given in the main text.

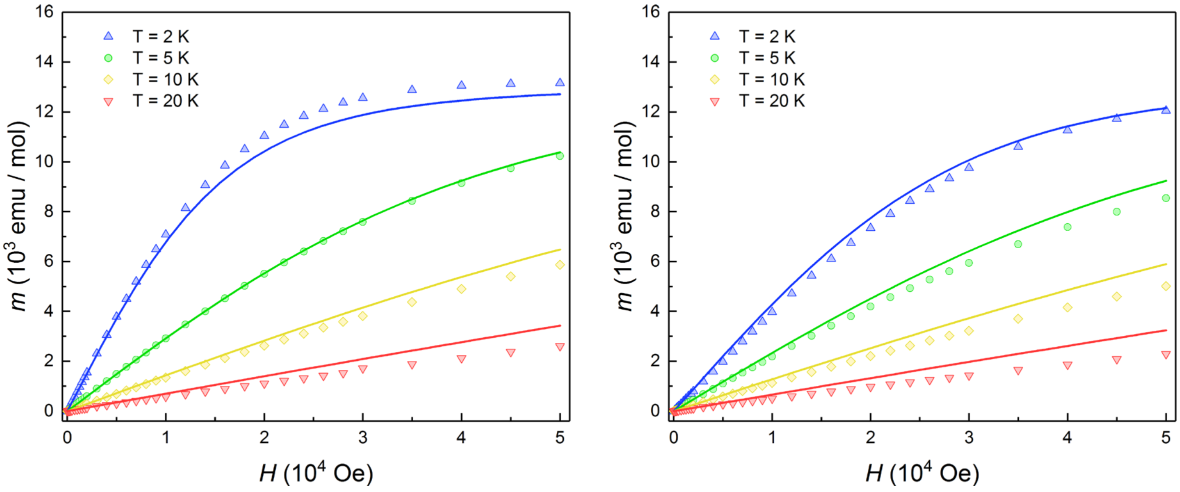

Figure S15. Magnetisation and magnetic susceptibility of a single crystal of **4** measured with the magnetic field applied along the maximum (left) and minimum (right) magnetisation orientations from Figure 9. Solid lines show the simulation of the magnetisation for the magnetic field applied along the cell *a* axis (left) and the cell *b* axis (right).

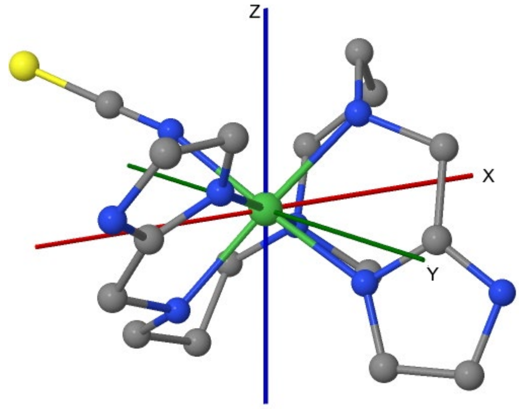

Figure S16. Orientation of the *D* tensor axes obtained from *ab initio* calculations, with *Z* (blue) the easy magnetization axis, *Y* (green) the intermediate magnetization axis and *X* (red) the hard magnetization axis.

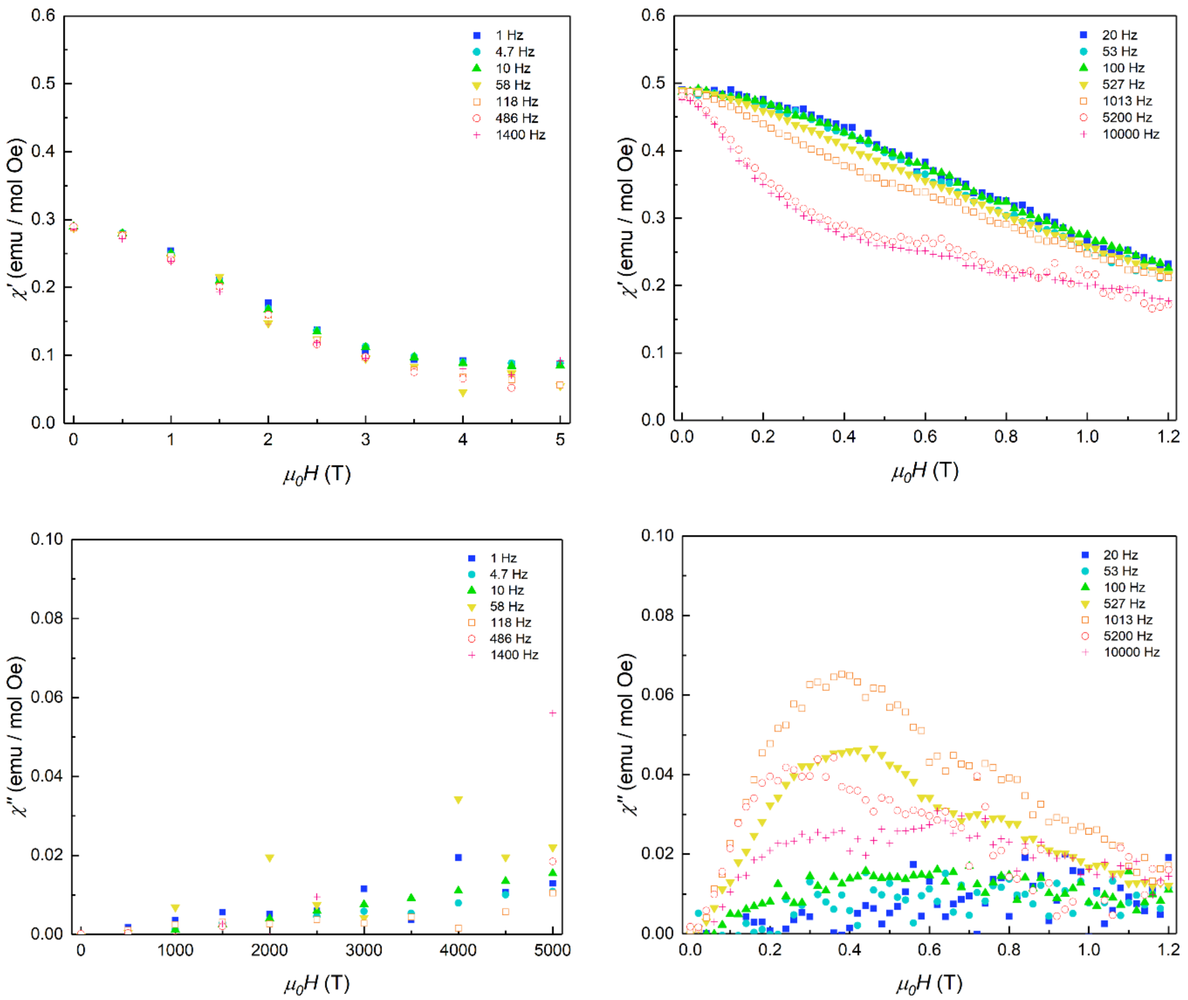

Figure S17. Ac susceptibility of powdered samples of **1** (left) and **4** (right) measured at T = 2 K as a function of magnetic field and for different frequencies $\omega/2\pi$ of the ac oscillating magnetic field. The top panels show the real component χ' and the bottom panels show the out-of-phase component χ''. The dependence of χ' on ω and the onset of a non-zero χ'' signal the existence of magnetic relaxation processes that 1) modify the magnetic response and 2) have relaxation time scales comparable to $1/\omega$. The results show that the irreversible susceptibility tends to vanish near zero field and that the spin relaxation is faster for complex **1**, which has a larger tunnelling splitting.

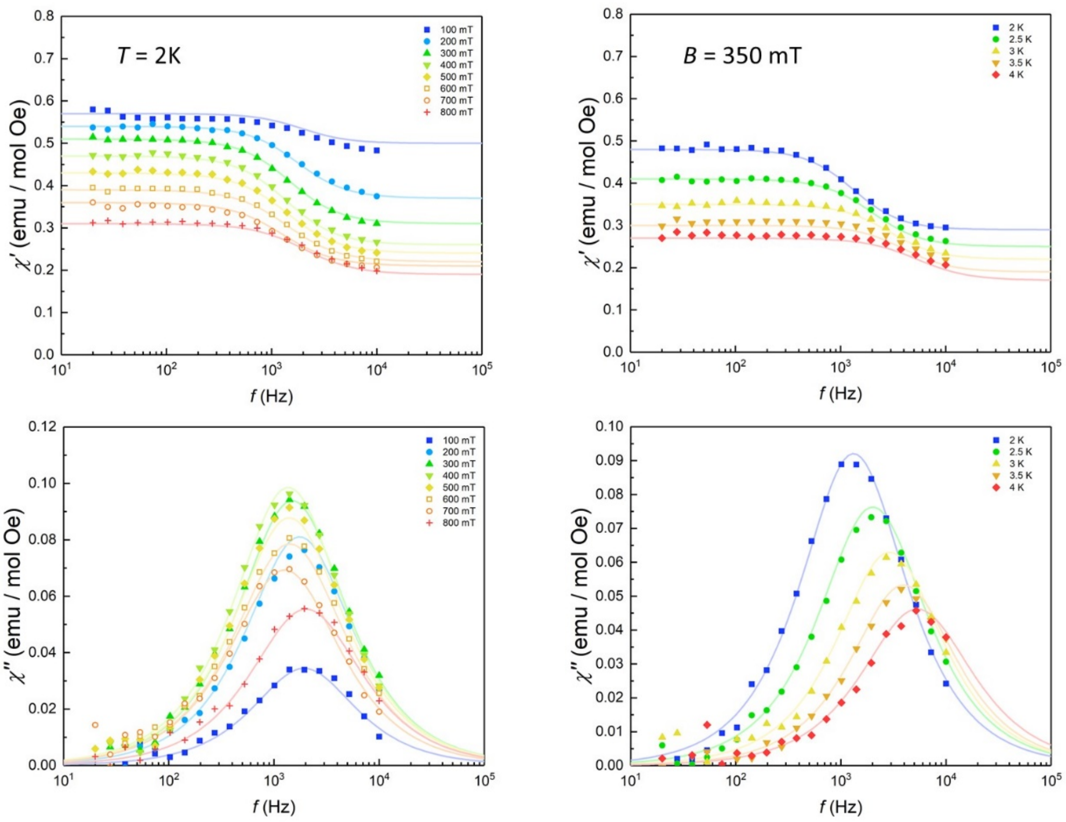

Figure S18. Frequency-dependent ac susceptibility of a single crystal of **4** measured at 2 K and different magnetic fields (left) and at *B* = 350 mT and different temperatures (right). The dc and ac magnetic fields were applied along the crystallographic axis. Top and bottom panels show, respectively, the in-phase χ' and out-of-phase χ" susceptibility components. The symbols are experimental data and the lines are least-square fits performed with Cole-Cole functions [6]

$$\chi'(\omega) = \chi_S + (\chi_T - \chi_S)\frac{1 + (\omega\tau)^{\beta}\cos\left(\frac{\pi\beta}{2}\right)}{1 + 2(\omega\tau)^{\beta}\cos\left(\frac{\pi\beta}{2}\right) + (\omega\tau)^{2\beta}}$$

$$\chi''(\omega) = (\chi_T - \chi_S)\frac{(\omega\tau)^{\beta}\sin\left(\frac{\pi\beta}{2}\right)}{1 + 2(\omega\tau)^{\beta}\cos\left(\frac{\pi\beta}{2}\right) + (\omega\tau)^{2\beta}}$$

where ω is the angular frequency, χ_T the isothermal susceptibility, χ_S the adiabatic susceptibility τ the average spin relaxation time and β describes a limited distribution of relaxation times (0.95-1 range).

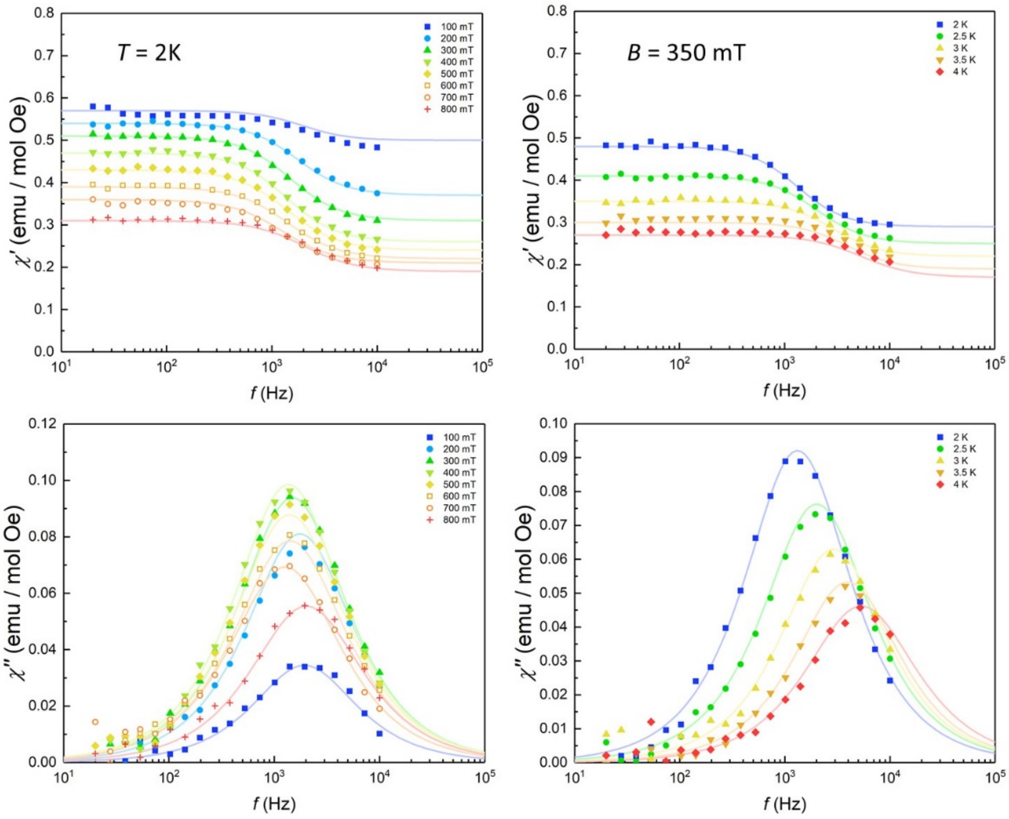

Figure S19. Frequency-dependent ac susceptibility of a powdered sample of **4** obtained by crushing the single crystal used in the measurements of Fig. S18 and mixing it in apiezon N grease. The measurements were then performed under identical conditions to those used for the single crystal. Top and bottom panels show, respectively, the in-phase χ' and out-of-phase χ'' susceptibility components. The symbols are experimental data and the lines are least-square fits performed with Cole-Cole functions [6]

$$\chi'(\omega) = \chi_S + (\chi_T - \chi_S)\frac{1 + (\omega\tau)^{\beta}\cos\left(\frac{\pi\beta}{2}\right)}{1 + 2(\omega\tau)^{\beta}\cos\left(\frac{\pi\beta}{2}\right) + (\omega\tau)^{2\beta}}$$

$$\chi''(\omega) = (\chi_T - \chi_S)\frac{(\omega\tau)^{\beta}\sin\left(\frac{\pi\beta}{2}\right)}{1 + 2(\omega\tau)^{\beta}\cos\left(\frac{\pi\beta}{2}\right) + (\omega\tau)^{2\beta}}$$

where ω is the angular frequency, χ_T the isothermal susceptibility, χ_S the adiabatic susceptibility τ the average spin relaxation time and β describes a limited distribution of relaxation times (0.95-1 range).

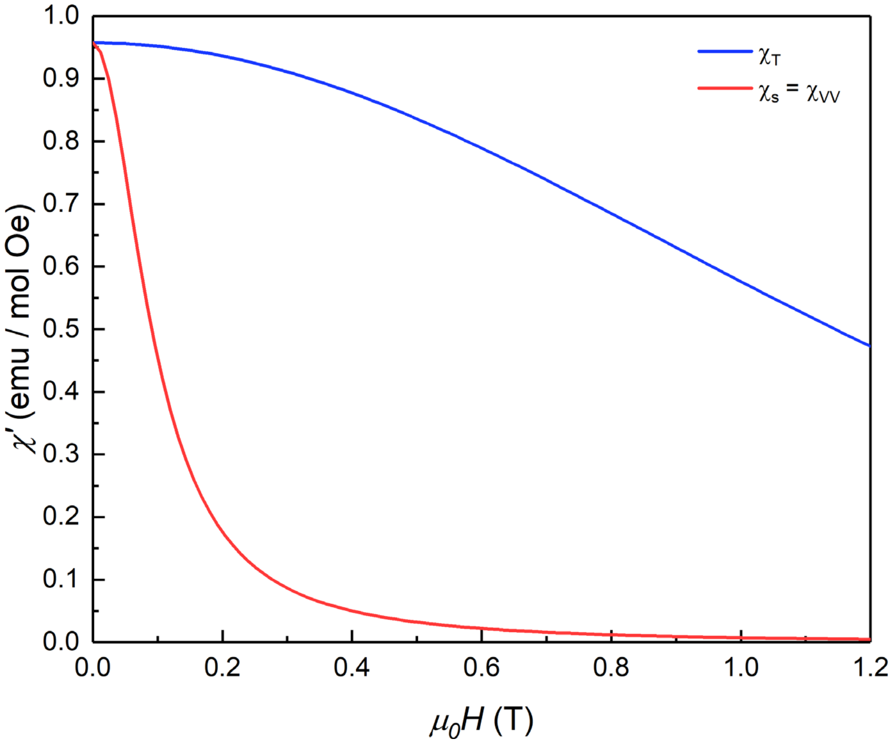

Figure S20. Magnetic field dependence of the equilibrium susceptibility χ_T and the reversible van Vleck susceptibility χ_S of complex **1** calculated from the spin Hamiltonian (1) with the anisotropy parameters given in the main text. The difference between the two represents the irreversible susceptibility, which is associated with phonon-induced transitions between different spin levels. Near the spin-clock transition at zero field, the spin wave functions are such that irreversible jumps between levels do not introduce any changes to the linear magnetic response. The ac susceptibility is then fully reversible and does not depend on frequency, even for spin lattice relaxation times $T_1 > 1/\omega$.

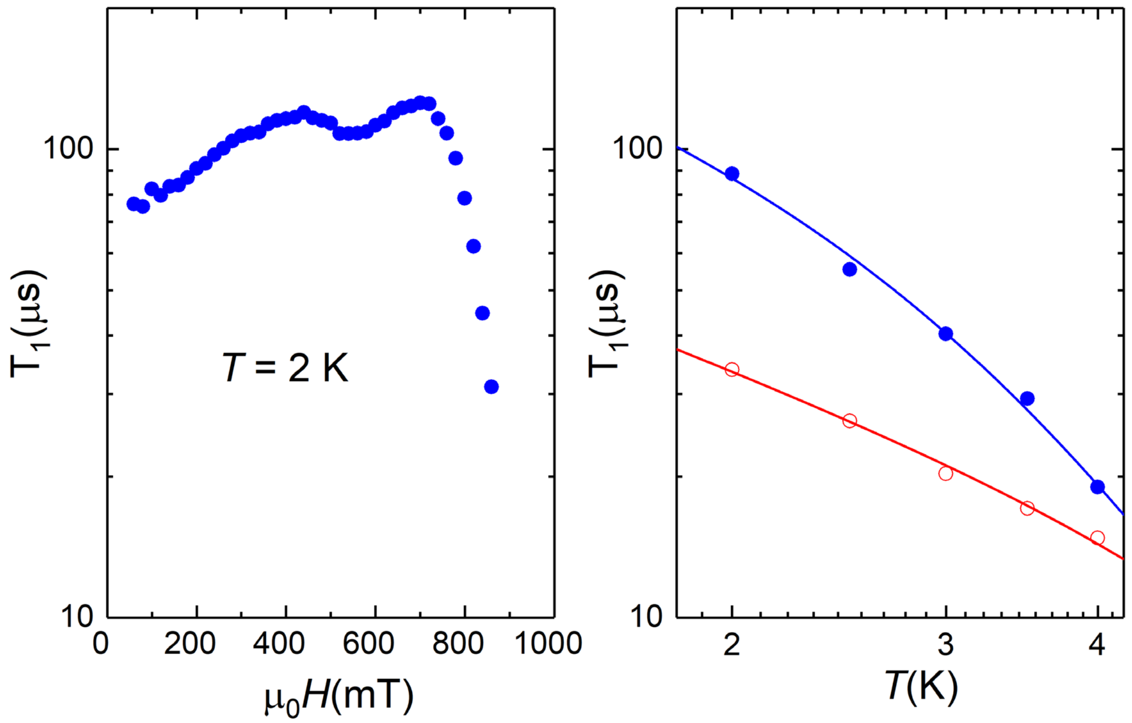

Figure S21. Spin-lattice relaxation time of **4** derived from frequency-dependent ac susceptibility experiments performed on a single crystal as a function of magnetic field at T = 2 K (left) and at two different magnetic fields as a function of temperature (right). The magnetic field was aligned perpendicular to the a and b crystallographic axes, thus approximately at 52.6 degrees from the magnetic anisotropy axis z (see Fig. 9 in the main text). The solid lines are fits that include direct and Raman relaxation processes (cf Eq. (5) of the main text).

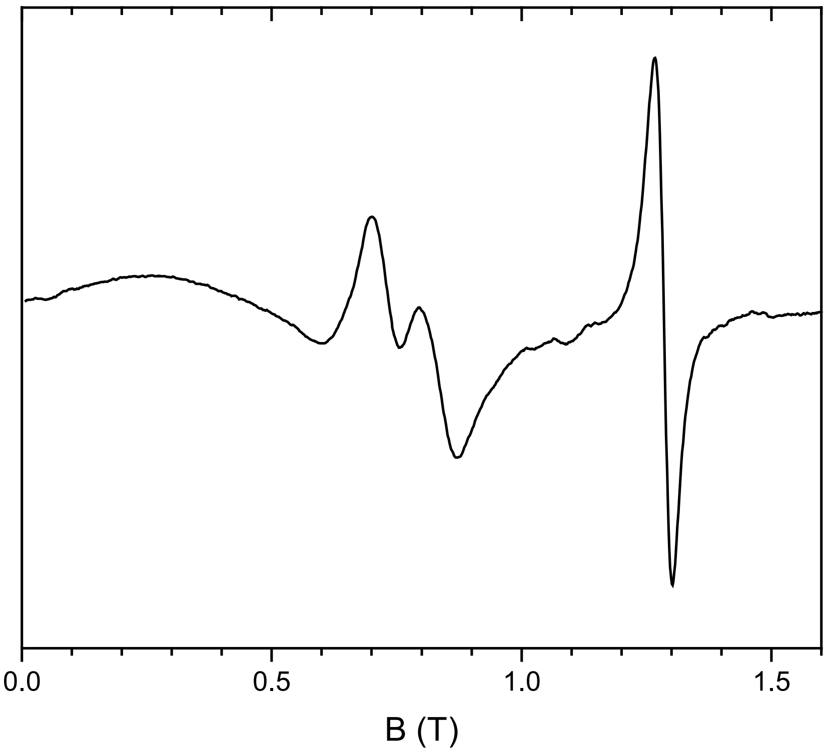

Figure S22. EPR spectrum of a large, but twinned, crystal of complex **4** measured at T = 2 K, a frequency of 110.4 GHz and a microwave power equal to 9 mW. A broad and structured signal is observed in the 0.4-1T field range as well as a well-resolved line at 1.2 T with a gaussian shape and a linewidth of about 340 G at 2 K, and much larger at 5 K. For these frequency and magnetic fields, the transitions observed must involve the two lowest lying spin levels.

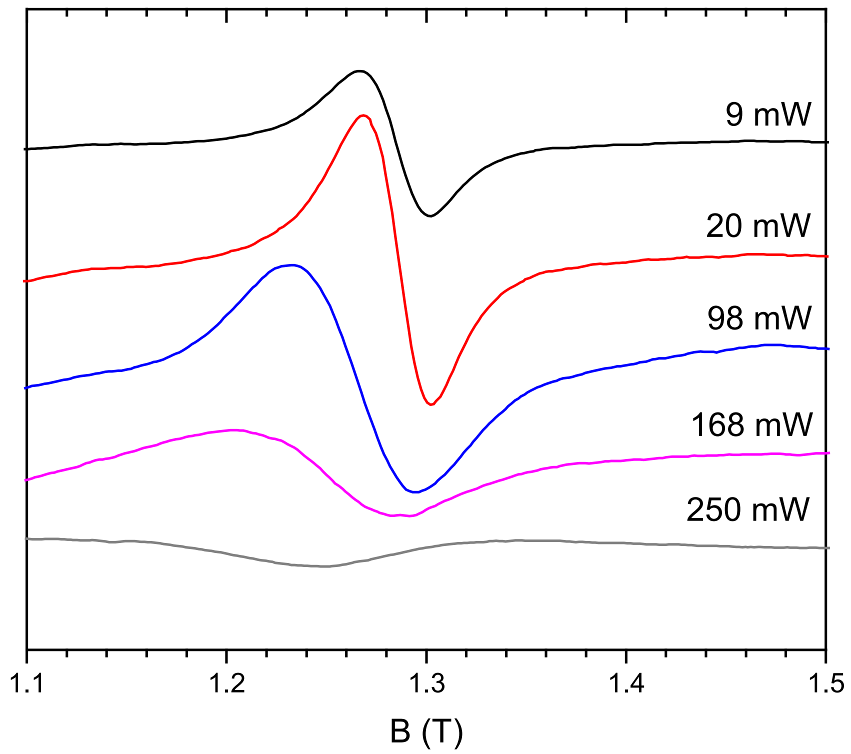

Figure S23. Change of the resonant signal observed near 1.2 T (cf Fig. S22) in the EPR spectrum of **1** as a function of the microwave power, for T = 2 K and a frequency equal to 110.4 GHz. With the increase of the applied power (up to about 270 mW), the resonance line broadens and its height goes through a smooth maximum for power in the range 120-140 mW. These saturation effects show that $(\gamma b)^2 T_1 T_2$, where $b \approx 0.01$-0.03 G is the microwave magnetic field amplitude, is no longer much smaller than 1. The resonance linewidth provides a lower bound for $T_2 > 1$-10 ns, although it is probably longer as the line shape shows the dominance of inhomogeneous broadening. The spin-lattice relaxation time T_1 can then be roughly estimated to be of the order of some tens of microseconds. More involved measurements are planned in order to better define T_1 (relying on longitudinal detection of the EPR signal) and eventually measure T_2.

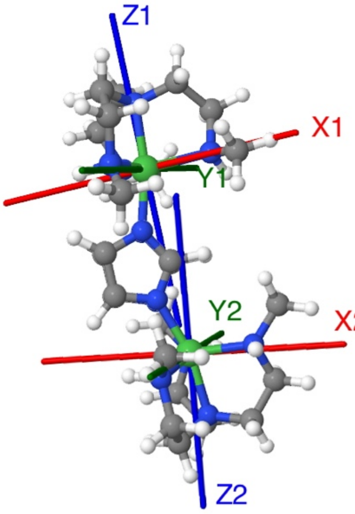

Figure S24. Schematic view of a Ni(II) binuclear complex with pentacoordinate geometry similar to that of **1** with the two Ni(II) having different spin Hamiltonian parameters (D_1, E_1 and D_2, E_2) and therefore different anisotropy tensor orientations [7].

4. References

[1] G. M. Sheldrick, SHELXS-97, Program for Crystal Structure Solution, University of Göttingen, Göttingen, Germany, **1997**.

[2] G. M. Sheldrick, *Acta Crystallogr. A* **2008**, *64*, 112-122.

[3] L. J. Farrugia, *J. Appl. Cryst*. **1999**, *32*, 837-838.

[4] R. Ruamps, L. J. Batchelor, R. Guillot, G. Zakhia, A. L. Barra, W. Wernsdorfer, N. Guihéry and T. Mallah, *Chem. Sci.* **2014**, *5*, 3418–3424.

[5] http://www.jcrystal.com/products/krystalshaper/

[6] K. S. Cole and R. H. Cole, *J. Chem. Phys.* **1941**, *9*, 341–352.

[7] F. El-Khatib, B. Cahier, M. Lopez-Jorda, R. Guillot, E. Riviere, H. Hafez, Z. Saad, J. J. Girerd, N. Guihery and T. Mallah, *Inorg. Chem.*, 2017, **56**, 10655-10663.
